# Support groups for dementia caregivers - Predictors for utilisation and expected quality from a family caregiver's point of view: A questionnaire survey PART I*

**DOI:** 10.1186/1472-6963-10-219

**Published:** 2010-07-28

**Authors:** Elmar Gräßel, Angelika Trilling, Carolin Donath, Katharina Luttenberger

**Affiliations:** 1 Medical Psychology and Medical Sociology, Clinic for Psychiatry and Psychotherapy, Erlangen University Hospital, Schwabachanlage 6, 91054 Erlangen, Germany; 2 The City of Kassel, Department of Social Services, 34112 Kassel, Germany

## Abstract

**Background:**

Support groups have proved to be effective in reducing the burden on family caregivers of dementia patients. Nevertheless, little is known about the factors that influence utilisation or quality expectations of family caregivers. These questions are addressed in the following paper.

**Methods:**

The cross-sectional study was carried out as an anonymous written survey of family caregivers of dementia patients in Germany. Qualitative and quantitative data from 404 caregivers were analysed using content analysis and binary logistic regression analysis.

**Results:**

The only significant predictor for utilisation is assessing how helpful support groups are for the individual care situation. Family caregivers all agree that psycho-educative orientation is a priority requirement.

**Conclusions:**

In order to increase the rate of utilisation, family caregivers must be convinced of the relevant advantages of using support groups. Support groups which offer an exchange of experiences, open discussion, information and advice meet the requirements of family caregivers.

## Background

Two challenges are identified in research into professional care for dementia patients. First: provision of appropriate care to the largest possible number of patients [1,2] and second optimisation of support for family caregivers. This is primarily a search for effective methods of support [3-5], and additionally, it should answer the question about how to motivate family caregivers and convince them to make use of support services. Our study deals with this question particularly with respect to support groups for family caregivers.

In support groups, family caregivers meet to exchange information under the guidance of an expert or an experienced family caregiver. They can discuss their stresses and strains, problems and most importantly, they experience emotional support [6,7]. Therefore support groups are a form of respite for family caregivers and have been proven in meta-analytical studies [5,8] and reviews [4,9] to have a highly significant effect (p < 0.001) on abilities and knowledge of the family caregiver and significant (p < 0.01) effects on subjective well-being and caregiver burden. Support groups for family caregivers are inferior in effectiveness to multicomponent training, but they are an effective, low-threshold and cost-effective offer. However, the degree of usage of support groups, according to international studies, is only between 4.8% und 14.0% [10-12]. In order to specifically influence this low level, it is important to know the predictors for utilisation and particularly those that represent the perspective of the family caregivers.

The question of which factors influence the utilisation of support groups has remained unanswered in publications to date. There are very few empirical studies about utilisation and only one about quality. Specific studies regarding support groups for dementia caregivers alone are absent as either several respite offers or community services were considered jointly or the results were not specifically related to dementia. Although fulfilment of quality standards is the main characteristic of a professional service, little is known about the specifics of effective intervention. Quality criteria have either been defined as expert standards [13] or based on the outcome variables in studies of family caregiver interventions [e.g. [14]]. In this context, support and respite for family caregivers are the most frequent aspirations. These recommendations are not aimed specifically at the dementia situation. They do not refer to a specific intervention, but are formulated for community services as a whole and do not respect the family caregivers' wishes. The quality of support groups as regards "user-friendliness" would be improved if the "customer's" - family caregiver's - concept of quality were systematically taken into consideration. To date no empirical study on the quality of support groups which include the quality wishes of dementia caregivers could be found in Medline^®^, PsychINFO^® ^or Cinahl^®^.

Based on the above reflections, our study has two main objectives:

The *first objective *is to determine which variables concerning the family caregiver's point of view influence the utilisation of support groups.

The *second objective *is to determine which quality characteristics of support groups from a family caregiver's point of view should be fulfilled, dependent on whether support groups have already been used ("user") or not ("non-user"). In an exploratory analysis, our study therefore shows predictors and quality criteria of family caregivers of dementia patients on support groups.

## Methods

### Design

The data base of the study is a written anonymous survey of family caregivers of dementia patients living in the community. The cross-sectional study was carried out in four regions in Germany - in Erlangen and district (Southern region), in Dortmund and district (West), in Kassel and district (North central) and in the Federal State of Brandenburg, specifically in the region around Potsdam (Northeast). Each study region had urban and rural areas with a minimum number of 250,000 inhabitants and therefore at least 2,500 dementia patients [15].

The survey packet consisted of a letter, the questionnaire, a stamped addressed envelope and an information brochure titled "Das Wichtigste über die Alzheimer-Krankheit" (The most important facts about Alzheimer's disease) [16]. The anonymity of the study in particular was emphasised in the letter. The objective was, on the one hand, to reach family caregivers who had no previous experience with support groups ("non-users") and on the other, to gain information from those who had already used or were using them ("users"). In order to reach the non-users, 200 questionnaires were to be distributed in each study region through the Medical Services of the Health Insurance offices at the initial appraisal of the patient's need for care under the long-term nursing care regulations. This procedure initiates allotment of the financial resources which allow the caregivers access to respite care. Hence, this recruiting pathway facilitated the contact to dementia caregivers, who in most cases had never used support groups. If one of the two care diagnoses made at the initial appraisal was "dementia," the survey packet was to be given to the family caregiver. In order to reach caregivers with experience of support groups, 300 questionnaires were to be distributed through the regional offices of the Alzheimer's Society and other caregiver counselling services. Using this recruiting pathway, there is a good chance that a family already using one service (in our study "caregiver counselling") might also have had some experience with other relief offers, such as support groups [12]. The recruitment period was limited to six months.

Of the 2,000 questionnaires (500 in each region) sent to the distributors, 404 were returned, giving a response of at least 20.2%. This response rate is based on the questionnaires which were sent to the distributors, not on those that actually reached the caregivers.

### Instruments

The 3-page questionnaire (see Additional File 1) was tested on 12 family caregivers for comprehensibility and acceptability in a pilot phase.

#### Quantitative data

Besides the socio-demographic variables (see Table 1), characteristics of the care situation and variables in connection with support groups were collected. The care situation is fundamentally characterised by the amount of care time required by the caregiver, whether he/she gets help from others and whether the patient has been classified within the health care insurance system. First support groups were described briefly. Then the questions "Do you know about support groups?" and "Have you ever used support groups" were to be answered dichotomously (yes/no). The assessment of the extent to which support groups are needed by the family caregiver in his/her care situation was carried out on a 5-step Likert Scale (from "0 = I don't need them" to "4 = I need them urgently") with the addition of "independent of whether you have previously used support groups or not". In addition, family caregivers were to grade the accessibility of support groups according to the three categories "Don't know", "Not easily accessible" or "Accessible".

**Table 1:** Sample characteristics of participating family caregivers and patients

Variable	Mean (SD) or Frequency in %
**Family caregiver**	

Age (years)	61.3 (11.9)

Gender (female)	73.3%

Education level	
- No school leaving certificate	0.8%
- Secondary school (9 years)	47.7%
- Vocational school (10 years)	30.7%
- Grammar school (13 years)	20.8%

Employed	28.6%

Relationship to patient	
- Spouse	43.8%
- Adult children	48.9%
- Others	7.3%

Place of residence (city)^a^	44.4%

Caregiver/patient sharing accommodation (apartment/house)	75.0%

Time for care per day (hours)	5.1 (4.7)

No caregiving help from others	34.8%

**Dementia patient**	

Age (years)	78.8 (9.1)

Gender (female)	63.6%

Level of care (health insurance)^b^	
- Not yet applied for	5.8%
- Applied for	19.2%
- Level 1	29.6%
- Level 2	31.6%
- Level 3	13.7%

Duration of dementia (years)	4.2 (3.3)

^a ^100,000 or more inhabitants (= city)

^b ^the higher the level, the higher the necessary help

#### Qualitative data

The data for qualitative analysis was collected using an open question: "Independent of whether you have already used support groups or not: What would you personally expect from a "good" support group?" The collection of data on the quality of support groups was carried out using open questions for two reasons: (a) there was no standardised, validated questionnaire which included all relevant quality aspects. (b) The "free recall" of one's own reflections represents the family caregiver's concept of quality without being influenced by extrinsic arguments.

### Description of the sample

The features of the family caregivers and their dementia patients can be seen in Table 1.

### Ethical Considerations

This is a cross-sectional written questionnaire study. The ethically relevant aspects of "voluntary disclosure of information" and "anonymity of answers" were dealt with in the accompanying letter, which was accepted by the Board of the regional Alzheimer's Society and presented to the Management of the German Alzheimer's Society for approval.

### Statistical procedure

#### Quantitative data analysis

The quantitative questions were answered by 97.9% of the 404 family caregivers on average. Binary logistic regression analysis was carried out to ascertain which variables significantly influence the utilisation of support groups. The dichotomous dependent variable was the "use" (code = 1) or "non-use" (0) of support groups. The coding of potential predictors is shown in the legend of Table 2. A multicollinearity test was carried out before the regression analysis in order to exclude confounded variables because of a significant correlation of moderate strength (r > 0.4). Therefore the following variables were not included in the multivariate regression analysis: relationship between caregivers and dementia patients, shared accommodation, employed caregivers, level of care (health insurance), accessibility of support groups. The explained variance in the regression model is quoted with Nagelkerke's R^2^. The significance of potential predictors was measured using Wald's coefficient (α = 0.05).

**Table 2:** Binary logistic regression analysis with use of support groups as dependent variable

	Regression coefficient β	Standard error	**Wald coeff**.	p	Odds ratio	95% Confidence interval for odds ratio	
						**Lower value**	**Higher value**

Gender of family caregiver^a^	-.294	.459	.411	.522	.745	.303	1.833

Gender of dementia patient^a^	-.461	.402	1.315	.251	.631	.287	1.386

Age of family caregiver	.001	.018	.003	.956	1.001	.967	1.036

Level of caregiver's education^b^	.421	.377	1.244	.256	1.523	.727	3.188

Age of dementia patient	-.011	.020	.287	.592	.990	.952	1.028

Help from others (informal or formal caregivers)^c^	.071	.356	.040	.842	1.074	.534	2.158

Duration of illness	.004	.004	.783	.376	1.004	.995	1.013

Hours per day spent on care	.039	.043	.814	.367	1.040	.955	1.132

Place of residence (city)^c^	-.334	.355	.888	.346	.716	.357	1.435

"Knowing" about support groups^c^	-21.489	3997.925	< .001	.996	< .001	< .001	.

"Need" for support groups	1.292	.175	54.253	< .001**	3.639	2.581	5.132

^a ^Gender: 0 = male, 1 = female

^b ^Level of caregiver's education: 0 = secondary school or no school leaving certificate, 1 = vocational school or grammar school

^c ^0 = yes, 1 = no

p: significance (** α ≤ 0.001)

#### Qualitative data analysis

The question about the quality of support groups was evaluated using Morgan's [17] content analysis method. In the first step, passages with the same or very similar content were paraphrased. In the second step, similar statements were merged into categories, modelled on the choice of words used by the interviewees, in order to keep the level of abstraction to a minimum. Two researchers independently undertook assignment to the individual categories. In individual cases of divergence, consensus had to be achieved. The frequency of citation of the categories is shown in Table 3. The design reflects general performance criteria of qualitative research, such as that the research process should follow clear rules, be exactly documented and that interpretation be well-founded. Finally, the individual conclusions were assigned by two independent researchers to the three quality categories, "Structure", "Process" and "Quality of results".

**Table 3:** Family caregivers' quality requirements - ranking of most frequent requirements (≥ 5%)

**Users **^**a**^: **Quality requirement (classification **^**b**^)	Number (%)	**Non-users **^**a**^: **Quality requirement (classification **^**b**^)	Number (%)
Exchanging experiences (P I)	74 (54)	Exchanging experiences (P I)	80 (65)

Communication: listening, candidness, understanding, taking everyone seriously (P II)	34 (25)	Communication: listening, candidness, understanding, taking everyone seriously (P II)	19 (15)

Information & tips (in general) (P I)	22 (16)	Psychological support: encouragement, consolation (P I)	16 (13)

Help & support (in general) (P I)	15 (11)	Information & tips about treating illness, dealing with dementia patients (P I)	12 (10)

Discussion (P I)	15 (11)	Feeling "not alone" (E II)	10 (8)

Psychological support: encouragement, consolation (P I)	14 (10)	Help & support (in general) (P I)	10 (8)

Information & tips about treating illness, dealing with dementia patients (P I)	13 (9)	Information & tips (in general) (P I)	9 (7)

Respite for family caregiver (E II)	9 (7)	Discussion (P I)	9 (7)

Support group meeting always at same time (S I)	8 (6)	Support group with leader (S I)	7 (6)

Feeling "not alone" (E II)	8 (6)	Respite for family caregiver (E II)	7 (6)

Talks by specialists (S I)	7 (5)		

^a ^164 family caregivers (40.6%) were users; of these 138 supplied details of quality requirements (≙ 100%)

230 family caregivers (56.9%) were non-users; of these 123 supplied details of quality requirements (≙ 100%)

10 family caregivers (2.5%) gave no details about utilisation

^b ^Classification of quality criteria:

• Structural quality (S):

I. Non-personal factors (S I)

II. Person-related factors (S II)

• Process quality (P):

I. Content aspects of procedure (What is done?) (P I)

II. Formal aspects of procedure (How is it done?) (P II)

• Quality of result (E):

I. Aims concerning dementia patients (E I)

II. Aims concerning family caregivers (E II)

## Results

At 70.1%, the existence of support groups is well known to family caregivers. Less than half (41.6%) of the respondents use them. In the assessment of how much this offer is needed, almost a third of the responding family caregivers need support groups very urgently (11.3%) or urgently (19.1%) while about two-fifths think they don't need them at all (24.1%) or hardly ever (17.1%) The remaining 28.4% were indifferent ("might need them to some extent").

Essentially, family caregivers have divided opinions about the accessibility of support groups. While about half the respondents (49.2%) stated that there was an accessible support group in their locality, 45.0% knew nothing about the distance to or the accessibility of their nearest support group. All in all, 5.8% of the respondents stated that support groups are not easily accessible to them.

### Regression Analysis

Binary logistic regression analysis showed that a significant regression model (χ^2 ^(11) = 213.143; p < .001), characterised by one significant predictor could be identified with an explained variance of 65.3% (R^2^) (Table 2). So 84.3% of the respondents can be correctly assigned to the categories "user" or "non-user". The probability of using support groups increases significantly when the degree of "need" increases. Utilisation is not associated with age, gender, level of education, rural versus urban living, help from others or to specific parameters of the illness such as the length of the patient's illness or the level of the patient's need for care.

We carried out a sensitivity analysis with all predictors neglecting the multicollinearity assumption. Again a significant regression model (χ^2 ^(17) = 226.922; p < .001) with a variance explanation of 70.8% (R^2^) resulted. The model differs from that described above in the number of significant predictors: Besides the degree of "need" (p < .001), the accessibility of support groups is a significant predictor for usage (p = .005). The probability of support group utilization is 77% lower for caregivers who do not know how to access the nearest support group and 50% lower for caregivers who say the nearest support group is not easily accessible.

### Content Analysis

The family caregivers' top three quality requirements concerning support groups overlap to a large extent for users and non-users. Top of the list for the majority of respondents was the wish to exchange caregiving experiences (54% and 65% respectively, Table 3). If both information categories, the global wish for information and the need for specific information about illness, treatments, and dealing with dementia patients are added together, information-seeking lies in second place (25% and 17% respectively). In third place, a form of group communication characterised by candidness and willingness to listen to each other is expected (25% and 15%).

## Discussion

Although the objectives of this study, utilisation and quality, are highly relevant for the further development of respite offers and community services, a systematic search of three databanks - Medline^®^, PsychINFO^®^, and Cinahl^® ^- shows that there has been little scientific research in these areas to date. Our study is the first which is concerned with support groups specifically for dementia caregivers and at the same time investigates the predictors for utilisation and indicators of the quality of support groups.

The sample of family caregivers was taken in four regions throughout Germany, which were suitable from the point of view of urban and rural with varying coverage of support groups. It was not, however, a representative sampling. By using various recruiting pathways, the medical services of Health Insurance companies, regional offices of the Alzheimer's Society and other caregiver counselling centres, it was possible to reach family caregivers with varying experience of support groups.

The response rate was more than 20%. This value corresponds exactly with the empirically-established response in anonymously written surveys of family caregivers who received no reward for answering [18,19]. However, a recruitment bias cannot be excluded. The frequencies of the individual quality criteria should therefore be seen as orientation values. But by using various recruiting pathways, it was possible to affirm that a substantial fraction of family caregivers had never used support groups previously, as was to be expected in a representative sample. The fact that over 50% were non-users has also been seen in other studies about various respite offers [10-12].

It can be confirmed that support groups are comparatively well known (awareness level 70%). The level of awareness is similar to the 59% seen in a study by Toseland et al. [12], hence the first pre-requirement for utilisation is fulfilled. Brodaty et al. [20] however report that the utilisation of respite offers by family caregivers is low, even though these are well known and even when the services are free of charge [21]. This means that there must be other, more significant variables that determine utilisation. In our study, the family caregivers' assessment of need of support groups ("I need them") is a highly significant predictor for using them.

In a study by Toseland et al. [22], it was demonstrated that the assessment of need is not a significant predictor for utilisation in the range of professional health and human services. However, it must be taken into consideration that this study is not specific to any one offer. Utilisation which depends on how well known the offer is, is significantly moderated by the assessment of need when the utilisation refers to a single service, as in our study. Here 30% said that they needed support groups urgently or very urgently. This percentage is three times as high as in Toseland et al. [12] (9.0%) and about twice as high as in a study by Colantonio et al. [23] (17.5%).

But the results of studies by Monahan et al. [24] and Burks et al. [25] concur with those presented here by regression analysis. In both studies, the assessment of the helpfulness of care groups - "I need them" - was shown as a significant predictor for the utilisation of support groups by family caregivers of dementia patients. So it can be reasoned that if there are plans to relieve as many family caregivers as possible, the service should not only be made known to family caregivers but the individual advantages of utilising support groups for himself/herself should also be pointed out. The results of our sensitivity analysis also suggest that it is of great importance to give information about how a support group can be accessed. This information should be given in a very practical manner, including a time-table of public transportation and a road-map.

Contrary to our study, Monahan et al. [24] showed that the probability of the family caregiver's using a support group increases as the dementia patients become older and if there is help from other persons. But in conformity with our study, neither the age and gender of the family caregivers and patients, nor the duration of illness were significant predictors for utilisation [25,26].

All in all, only 5.8% of respondents reckoned that support groups were not readily accessible to them. This result is comparable to published results (13.3%) [12], where relatively low rates of poor accessibility to support groups are reported. Lack of knowledge about the nearest support group is, however, more frequent - 45% in our study and 41% in Toseland et al. [12]. It must be taken into consideration that in other studies, easy access to respite offers is positively associated with utilisation [21,22] and that in our study and in Toseland et al. [12], about half of respondents knew nothing about the accessibility of their nearest support group. Therefore, another practical objective should be to give clear instructions about the location of the nearest support group and how to get there.

Although multi-component trainings are superior in their effectiveness, and therefore are often investigated [see for example [27,28]], positive effects on caregivers' abilities and knowledge, burden and well-being through support groups could be shown in reviews [9] and in meta-analytical studies [8]. Despite this, little is known about the specifics of effective intervention. An empirical study about quality criteria for support groups could not be found in Medline^®^, PsychINFO^® ^or Cinahl^®^. Just one single study that documented the experience of 18 dementia family caregivers in group counselling sessions refers to the importance of the aspects "to talk about anything" and "to be understood by the others" using semi-structured interviews [29].

In our study with 404 responding family caregivers of a dementia patient, there is a pattern of agreement on the most frequent quality requirements, independent of whether the family caregivers already had attended support groups or not. Support groups should therefore aim for an intensive exchange of experiences which is characterised by the frankness of the members. Information and tips about dementia, treatments and dealing with dementia patients are often required.

This study introduces the family caregivers' views into the scientific discussion about quality standards for support groups for dementia caregivers, so that the extent of "unmet needs" [30] can be reduced. This is important because consideration of the family caregivers' quality requirements reduces the barrier to utilisation [31].

## Conclusions

In order to increase the utilisation of support groups, all family caregivers should not only be informed about the existence and accessibility of such offers but also about the advantages to themselves. This includes information about the location and accessibility of the nearest support group. In consequence, caregivers would have detailed information on which to base a decision concerning their need for support groups. Furthermore, support groups for dementia caregivers should concentrate primarily on moderated discussion of caregiving experiences. They should integrate psycho-educational elements, particularly information about the illness and available treatments as well as tips on how to deal with dementia patients. Regular utilisation by family caregivers who need this support offer can be realised by fulfilling "customer wishes".

## Competing interests

The authors declare that they have no competing interests.

## Authors' contributions

EG designed the study and its questionnaires, supervised data collection, was responsible for the interpretation of data and took part in writing the paper. AT assisted with designing the study, organised the data collection and gave important hints for interpretation and discussion. CD designed the study, was responsible for the statistical design and data analysis and took part in writing the paper. KL participated in its design and statistical analysis, took part in the interpretation and helped to draft the manuscript. All authors read and approved the final manuscript.

## Pre-publication history

The pre-publication history for this paper can be accessed here:

http://www.biomedcentral.com/1472-6963/10/219/prepub

## Supplementary Material

Additional file 1 **Questionnaire**. A word document including the questionnaire which was used to investigate knowledge and perception of support groups by caregivers of dementia patients. Click here for file
