# Supplementary material for: Support groups for dementia caregivers - Predictors for utilisation and expected quality from a family caregiver's point of view: A questionnaire survey PART I*
Source: BMC Health Serv Res. 2010 Jul 28;10:219. doi: 10.1186/1472-6963-10-219 (PMC2922206; doi:10.1186/1472-6963-10-219)
Supplement: Additional file 1 — Questionnaire. A word document including the questionnaire which was used to investigate knowledge and perception of support groups by caregivers of dementia patients. [file 1472-6963-10-219-S1.DOC]

# **Questionnaire for people who care for a dementia patient at home**

**1. Do you know about the support groups for family caregivers?**

(In support groups, family caregivers meet to exchange information under the guidance of an expert or an experienced family caregiver.)

 Yes  No

1. **How badly do you need the support groups in your care situation?**

**(independent of whether you knew of support groups previously or are already using the service or have used it in the past).**

| Don't need it | Hardly need it | Need it to some degree | Need it urgently | Need it very urgently |
| --- | --- | --- | --- | --- |
|  |  |  |  |  |

1. **To your knowledge, where is the nearest possibility for you to participate in a support group?**

 I don’t know

 Not easily accessible

 Accessible

1. **Have you ever used a support group for family caregivers?**

 Yes  No

- 1. If yes, how much respite did you obtain by participating in the support group with respect to dealing with the person for whom you care?

| Not at all | A little bit | Moderate respite | Quite a bit | A lot |
| --- | --- | --- | --- | --- |
|  |  |  |  |  |

- 1. Independent of whether you have ever used a support group or not: What would you personally expect from a "good" support group?

A good support group ………………………………………………………….

………….....................................................................................................

....................................................................................................................

....................................................................................................................

**Personal details:**

1. How old are you? ........... (Years)
2. Gender:  Female  Male

3. Education:

 Didn't finish school

 Secondary school

 Vocational school

 Grammar school

4. Are you employed at the moment?

 Yes  No

1. What is your relationship to the patient?

The patient is my:

 Spouse  Father

 Mother  Father-in-law

 Mother-in-law  Other: ..................................

1. How many inhabitants has the place where you live?

 under 1,000 inhabitants

 1,000 - 10,000 inhabitants

 10,000 - 100,000 inhabitants

 over 100,000 inhabitants

1. Do you live in the same house /apartment as the patient?

 Yes  No

**Patient's details:**

1. How old is the patient? ........... (Years)
2. Gender:  Female  Male
3. Which level of care (Health Insurance) has the patient?

 not applied for

 applied for

 Level I

 Level II

 Level III

**Details of care situation:**

1. Do you get any help with the care from family members, relatives, friends, acquaintances or neighbours?

 Yes  No

1. When was the dementia first diagnosed?

................. (Year)

1. How much time on average do you spend on care tasks in 24 hours?
   (e.g. personal hygiene, toilet, feeding etc.)

............ (hours)

**Many thanks for your co-operation**

Please use the stamped addressed envelope for your reply.
